# The Relationship between Serum Osteocalcin Concentration and Glucose Metabolism in Patients with Type 2 Diabetes Mellitus

**DOI:** 10.1155/2013/842598

**Published:** 2013-03-04

**Authors:** Qingqing Wang, Beibei Zhang, Yulan Xu, Hongdi Xu, Nan Zhang

**Affiliations:** ^1^Department of Endocrinology, Sir Run Run Shaw Hospital, College of Medicine, Zhejiang University, Hangzhou 310016, China; ^2^Department of Endocrinology, Zhejiang Qingchun Hospital, Hangzhou 310016, China

## Abstract

To study the correlations between serum osteocalcin and glucose metabolism in patients with type 2 diabetes, 66 cases were collected to determine total osteocalcin, undercarboxylated osteocalcin, fasting blood glucose, fasting insulin, and HbA1c. Osteocalcin concentrations were compared between groups of different levels of HbA1c, and parameters of glucose metabolism were compared between groups of different levels of total osteocalcin and undercarboxylated osteocalcin. The relationship between osteocalcin and parameters of glucose metabolism was also analyzed. We found that the total osteocalcin concentration of high-HbA1c group was significantly lower than that of low-HbA1c group. The fasting blood glucose of low-total-osteocalcin group was significantly higher than that of high-total-osteocalcin group in male participants, while the fasting blood glucose of low-undercarboxylated-osteocalcin group was significantly higher than that of high-undercarboxylated-osteocalcin group in all participants and in male participants. Total osteocalcin was inversely correlated with HbA1c, and undercarboxylated osteocalcin was inversely correlated with fasting blood glucose. However, no significant correlation was found between osteocalcin and HOMA-IR. Total osteocalcin was an independent related factor of HbA1c level. In summary, decreased serum total osteocalcin and undercarboxylated osteocalcin are closely related to the exacerbation of glucose metabolism disorder but have no relations with insulin resistance.

## 1. Introduction

Osteocalcin (OC) is a kind of noncollagenous protein which is synthesized and secreted by osteoblast. OC consists of undercarboxylated osteocalcin (ucOC) and fully carboxylated osteocalcin (cOC). cOC is formed after the glutamic acid residues of OC on the 17th, 21st, and 24th sites are carboxylated by vitamin K-dependent carboxylase. Osteocalcin with noncarboxylated glutamic acid residues is named as ucOC. Physiological functions of OC include maintaining normal bone mineralization, suppressing abnormal hydroxyapatite formation, and slowing down growth cartilage mineralization [1].

Previous human studies demonstrated that poorly controlled blood glucose could lead to reduced serum OC level in diabetic patients, while serum OC increased after blood glucose was well controlled [2]. These data indicated that changes of glucose metabolism could influence OC levels. Recent animal studies discovered that OC could reduce blood glucose, promote insulin secretion, and increase insulin sensitivity [3], indicating an important role of OC in the glucose metabolism regulation. Therefore, the theory that OC and glucose metabolism influenced each other was proposed.

To date, the evidences for OC influencing glucose metabolism were limited compared to those for glucose metabolism affecting OC. Whether OC affects energy metabolism still remains unclear in human. Moreover, ucOC was proved to play a leading role in promoting insulin synthesis and improving insulin resistance in animals [3], while several clinical studies suggested that cOC, rather than ucOC, played a more remarkable role in affecting insulin sensitivity in the human body [4, 5], which indicated that different components of OC might have different functions in human beings and animals. Therefore, accumulating data of ucOC and cOC, as well as total osteocalcin (tOC) is important to further discover the mechanisms of osteocalcin. However, data of current studies are mainly about tOC, while data of ucOC or cOC are rare. This study aims to further investigate the influence of different OC components on glucose metabolism.

## 2. Materials and Methods

### 2.1. Objects and Grouping

We chose patients with type 2 diabetes mellitus (DM) who were admitted to Sir Run Run Shaw Hospital from March 2011 to March 2012 as research objects, referring to the 1999 WHO diagnostic criteria of diabetes mellitus. Patients with the following treatments or complications were excluded from the study: (i) acute diabetic complications, (ii) insulin treatment, (iii) using agents such as thiazolidinediones, statin, vitamin K, warfarin, vitamin D, calcium supplement, bisphosphonates, vitamin A, and hormones, (iv) bone diseases such as bone tumors, osteoporosis, and fracture, (v) nephropathy, liver dysfunction, kidney dysfunction, ovarian tumor, thyroid diseases, parathyroid diseases, and other endocrine diseases, and (vi) infection, trauma, major operation, and other stress. This study was approved by the medical ethic committee at Sir Run Run Shaw Hospital. All participants signed written informed consent.

First, the patients were divided into three groups, Group HbA1c-H (HbA1c ≥ 9%), Group HbA1c-M (7% ≤ HbA1c < 9%), and Group HbA1c-L (HbA1c < 7%) [6], as HbA1c < 7% indicated well-controlled blood glucose and HbA1c ≥ 9% indicated marked hyperglycemia [7]. TOC, ucOC, and other indexes were compared among the three groups. Then, the patients were divided into two groups according to the average level of tOC, Group tOC-H and Group tOC-L. Indexes of glucose metabolism were compared between the two groups. At last, the patients were divided into two groups according to the average level of ucOC, Group ucOC-H and Group ucOC-L. Indexes of glucose metabolism were also compared between the two groups. 

### 2.2. Specimen Collection

The age, gender, medication history, menstruation, course of diabetes mellitus, and complications of each patient were recorded. The liver and kidney functions, 24-hour urine microalbumin, creatinine clearance rate, bone mineral density, and thyroid function were also examined. Then, the research objects were chosen according to the inclusion and exclusion criteria. The venous blood of every research object was collected after 12-hour overnight fasting state, and levels of fasting blood glucose, fasting insulin, HbA1c, serum creatinine (sCr), blood calcium (Ca), bone-specific alkaline phosphatase (Ostase), and parathyroid hormone (PTH) were detected in the clinical laboratory of Sir Run Run Shaw hospital. Meanwhile, 4 mL of residual blood sample was collected and centrifuged, and the serum was kept in the fridge at the temperature of −20°C for tOC and ucOC detection. Homeostasis model assessment for beta-cell function (HOMA-*β*) was calculated to assess the basal insulin secretion of pancreatic beta cells. The formula was HOMA-*β*(%) = fasting  insulin × 20/(fasting blood glucose − 3.5). Homeostasis model assessment for insulin resistance (HOMA-IR) was calculated to estimate insulin sensitivity. The formula was HOMA-IR = fasting blood glucose × fasting insulin/22.5. The unit of fasting blood glucose was mmol/L, and the unit of fasting insulin was *μ*U/mL. The height and weight of the research objects were measured by a special-assigned person, and the body mass indexes (BMI) were calculated. 

### 2.3. Assay of OC

TOC was detected by radioimmunoassay. The kit was provided by Beijing Atom HighTech Co., Ltd. The product batch number was 201203. UcOC was detected by enzyme-linked immunosorbent assay. The kit was provided by the United States R&D company. The product batch number was 10-25-931.

### 2.4. Statistical Method

Measurement data that conformed to the normal distribution were compared by Student's *t*-test. Data which did not conform to the normal distribution were converted to the normal distribution and compared. Data of sex composition ratio and postmenopausal women composition ratio were compared by chi-square test. The correlations of OC and other indexes were analyzed by Pearson correlation analysis. The correlations of HbA1c and other indexes were analyzed by Pearson correlation analysis and stepwise multiple regression analysis. *P* < 0.05 by two-tailed test was considered as significantly different between the two groups. Data were processed with the software package of SPSS 11.5.

## 3. Results

A number of 66 cases were finally included, consisting of 46 men and 20 women. Seventeen of the women were postmenopausal. The principal characteristics of the objects were displayed in Table 1. The average age of the female objects was older than that of the male objects, but there was no significant difference between the average ages of the male and all the objects. The average HOMA-*β* of the female objects was higher than that of the male objects.

### 3.1. Comparisons between Different Groups

Several parameters of bone and glucose metabolism were compared between different groups. Comparisons among Groups HbA1c-H, HbA1c-M, and HbA1c-L were displayed in Table 2. Comparisons between Groups tOC-H and tOC-L were displayed in Table 3. Comparisons between Groups ucOC-H and ucOC-L were displayed in Table 4.

### 3.2. Pearson Correlation Analysis

**Figure 1:**
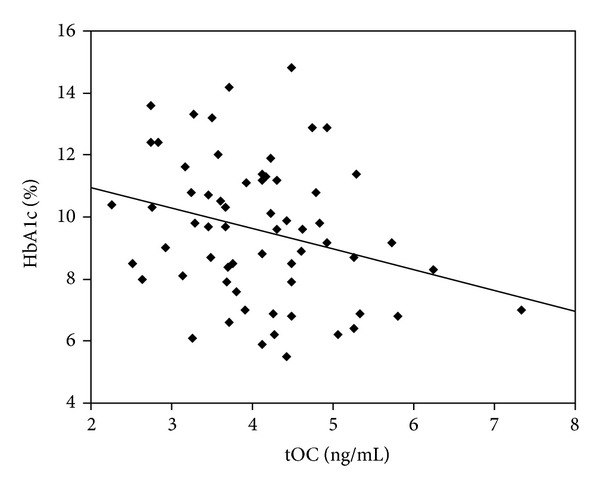
Negative correlation between tOC and HbA1c (*r* = −0.277, *P* = 0.028).

**Figure 2:**
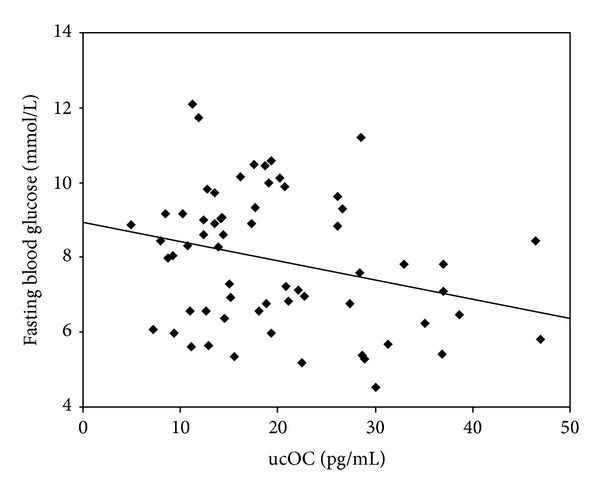
Negative correlation between ucOC and fasting blood glucose (*r* = −0.278, *P* = 0.027).

The Pearson correlation analysis showed that tOC was positively correlated with Ostase, negatively correlated with HbA1c (Figure 1), and not related with fasting blood glucose, fasting insulin, HOMA-*β*, HOMA-IR, or ucOC. ucOC was negatively correlated with fasting blood glucose (Figure 2), but not related with age, HbA1c, fasting insulin, HOMA-*β*, HOMA-IR, or tOC.

### 3.3. The Stepwise Multiple Regression Analysis of HbA1c and Other Indexes

We took HbA1c as a dependent variable. Age, BMI, course of DM, Ostase, Ca, sCr, fasting blood glucose, fasting insulin, HOMA-*β*, HOMA-IR, ucOC, and tOC were selected as independent variables. The stepwise multiple regression analysis showed that Ostase and tOC were both the independent relevant factors affecting HbA1c levels, whether the female objects were excluded or not (Table 5).

## 4. Discussion

### 4.1. tOC and Glucose Metabolism

The previous human studies demonstrated that poorly controlled blood glucose could lead to reduced serum OC level in diabetic patients, while serum OC would increase after blood glucose was controlled [2]. The animal model study conducted by Lee et al. found that OC gene knockout (OC^−/−^) could lead to elevated blood glucose and insulin levels, as well as decreased insulin sensitivity [3], suggesting that OC could in turn regulate glucose metabolism. Does OC have the similar function in human body? The answer is uncertain, because the vast majority of the present human studies are cross-sectional studies.

Some studies on Caucasian and Asian populations [8–10] showed that tOC level was significantly negatively correlated with fasting blood glucose, fasting insulin, and HOMA-IR. However, several Chinese studies showed that tOC was not related to HOMA-IR [11, 12]. This study found that tOC level of Group HbA1c-H was significantly different from that of Group HbA1c-L, tOC was negatively correlated with HbA1c and had no relationship with fasting blood glucose, fasting insulin, or HOMA-IR, and tOC was an independent associated factor of HbA1c. After excluding all the female patients, we observed that the fasting blood glucose of Group tOC-L was significantly different from that of Group tOC-H. These results suggested that decreased serum tOC was related with long-term hyperglycemia but had little impact on insulin resistance. The results of this study are similar to those of the other two Chinese studies, but different from those of the foreign studies. We think this may be caused by the differences in study populations (with different disease background and ages), races, genes, sunlight exposure, and diet habits.

Moreover, this study showed that HbA1c was positively correlated with Ostase while negatively correlated with blood calcium. The correlations disappeared after excluding the female objects. As we all know, the estrogen concentrations in postmenopausal women are lower. As a result, the lowered activity of vitamin D results in the decrease of blood calcium, the enhancement of bone turnover, and the increase of Ostase level. Therefore, we consider the phenomenon mentioned above may be related to the estrogen interference. However, the sample size became smaller after excluding the female objects, which limited the credibility of this study. Further researches are necessary to confirm the hypothesis above.

### 4.2. ucOC and Glucose Metabolism

Both *in vivo* and *in vitro* studies have indicated that it was probably the ucOC ingredient that played the leading role in lowering blood sugar, promoting insulin synthesis, and improving insulin resistance [3, 13]. Human studies in recent years have accumulated a small amount of ucOC data. These date, unlike the conclusions of animal studies, suggest that ucOC may have some association with insulin secretion and decreased blood glucose but have no relationship with insulin sensitivity [14–16]. This study found that the fasting blood glucose of Group ucOC-L was significantly higher than that of Group ucOC-H, and ucOC was negatively correlated with fasting blood glucose. But we failed to find any correlations between ucOC and HbA1c, fasting insulin, HOMA-*β*, HOMA-IR, and other indicators of glucose metabolism. These results suggested that ucOC decrease was related to the fasting blood glucose increase, but not related to the changes of insulin secretion and insulin resistance, partly in line with the conclusions of the aforementioned human studies. 

Whether ucOC is able to improve insulin sensitivity is the main disagreement between animal studies and human studies. As a matter of fact, the human studies cited above and this study are all cross-sectional studies, which are unable to clarify the causality of serum osteocalcin variation and glucose metabolism changes. Two retrospective studies of a clinical trial [17] may be more persuasive. One study found that elevated cOC level and reduced ucOC% (the percentage of ucOC in OC) caused by vitamin K supplement might improve insulin resistance but had no effects on the basal secretion of insulin [4]. The other one also found that cOC had a greater impact on insulin sensitivity than ucOC [5]. These human studies inspire us to further investigate the mechanisms of different OC components on the regulation of glucose metabolism in humans. Perhaps it is the cOC rather than ucOC that plays a leading role in regulating insulin sensitivity in humans, which is distinctive from the mechanism in animals.

In addition, this study also found that HOMA-IR of Group ucOC-L was significantly higher than that of Group ucOC-H after excluding the female objects. But we were unable to draw the conclusion that reduced ucOC levels were related to the enhanced insulin resistance. Since the BMIs of Group ucOC-H and Group ucOC-L were significantly different after excluding the female patients, we considered the higher HOMA-IR level in Group ucOC-L was related to its higher mean BMI.

## 5. Deficiencies and Limitations

Most of the current studies about the relationship between osteocalcin and glucose metabolism only detected tOC levels. Data of ucOC are little. Our study investigates not only the serum tOC concentrations, but also the ucOC levels, accumulating more research data to help further explore the mechanism of osteocalcin in glucose metabolism. However, there are some deficiencies and limitations in this study. First, the sample size is small, and there are no healthy individuals served as normal controls. However, it is worth mentioning that specimens of healthy individuals are being collected as controls at present. Secondly, the correlation analysis showed that there was no linear relationship between tOC and ucOC, partly because of the different kits used for determining tOC and ucOC. Gundberg et al. thought that serum ucOC was greatly impacted by serum tOC. Therefore they recommended ucOC% to reflect the serum ucOC level [18]. In this study, we could not calculate ucOC% because of the difference in kits, which is a disadvantage for the accuracy of analysis. Thirdly, we did not determine the serum cOC levels. Some human studies showed that cOC had a greater influence than ucOC on insulin sensitivity. Therefore, if tOC and cOC levels and ucOC% are simultaneously determined, the effects of different osteocalcin components on glucose metabolism may be further explored. Lastly, this research is a cross-sectional study, which cannot reveal the causal relationship of glucose metabolism and bone metabolism. Longitudinal studies are needed to further confirm whether the change of osteocalcin level, as an initiating factor, has some impact on insulin secretion and sensitivity.

## 6. Conclusions

By detecting and comparing the serum osteocalcin levels and glucose metabolism indicators of 66 patients with type 2 diabetes, we found that the tOC levels of patients with higher HbA1c were lower than those of the patients with well-controlled blood glucose levels, the fasting glucose levels were higher in male patients with lower tOC levels, tOC and HbA1c were significantly negatively correlated, and tOC was the independent associated factor affecting HbA1c level. These results indicated that there was a significant correlation between decreased tOC level and aggravating glucose metabolism disorder. In addition, we also collected data of ucOC, from which we found significantly higher fasting blood glucose levels in patients with lower ucOC levels. ucOC level was significantly inversely correlated with fasting blood glucose but was not associated with HOMA-IR. These results indicated that ucOC reduction may be associated with the aggravation of glucose metabolism disorders, but no influence of ucOC on insulin sensitivity could be proved.

## Figures and Tables

**Table 1:** The principal characteristics of the research objects.

Parameters	All the objects (*n* = 66)	The male objects (*n* = 46)	The female objects (*n* = 20)
Age (year)	51.9 ± 13.3	49.8 ± 13.0	57.1 ± 12.0^#^
Height (cm)	164.76 ± 8.56	168.80 ± 6.45*	156.10 ± 5.49^∗∗##^
Weight (kg)	65.92 ± 11.50	69.74 ± 9.97	57.45 ± 9.79^∗∗##^
BMI (kg/m^2^)	24.16 ± 3.09	24.43 ± 2.91	23.48 ± 3.29
Course of DM (month)	67.8 ± 53.7	56.2 ± 49.9	92.3 ± 54.6^#^
sCr (*μ*mol/L)	72.4 ± 12.5	78.0 ± 11.0*	60.6 ± 9.2^∗∗##^
Ca (mmol/L)	2.37 ± 0.13	2.37 ± 0.13	2.37 ± 0.11
Ostase (*μ*g/L)	13.98 ± 4.09	13.34 ± 3.21	15.33 ± 5.35
PTH (ng/L)	36.41 ± 10.95	38.69 ± 15.53	35.89 ± 10.00
HbA1c (%)	9.6 ± 2.3	9.3 ± 2.3	9.9 ± 2.1
Fasting blood glucose (mmol/L)	7.92 ± 1.81	8.11 ± 1.64	7.90 ± 2.61
Fasting insulin (*μ*IU/mL)	6.04 ± 3.06	5.74 ± 3.16	6.56 ± 2.60
HOMA-*β* (%)	33.56 ± 22.99	28.37 ± 15.82	43.74 ± 31.91^#^
HOMA-IR	2.13 ± 1.27	2.10 ± 1.36	2.23 ± 0.98
tOC (ng/mL)	4.08 ± 0.95	4.03 ± 0.91	4.31 ± 1.06
ucOC (pg/mL)	19.91 ± 9.76	21.04 ± 12.66	21.16 ± 10.53

**P < 0.01 when compared with all the objects. *P < 0.05 when compared with all the objects. ^##^ *P* < 0.01 when compared with the male objects.^ #^ *P* < 0.05 when compared with the male objects.

**Table 2:** Comparisons among Groups HbA1c-L, HbA1c-M, and HbA1c-H.

Parameters	HbA1c-L (*n* = 11)	HbA1c-M (*n* = 16)	HbA1c-H (*n* = 36)
Age (year)	46.5 ± 9.6	54.5 ± 11.9	52.5 ± 14.6
BMI (kg/m^2^)	24.22 ± 2.54	24.25 ± 3.37	24.11 ± 3.20
sCr (*μ*mol/L)	71.1 ± 9.9	73.8 ± 12.2	72.1 ± 13.6
Ca (mmol/L)	2.42 ± 0.14	2.39 ± 0.13	2.35 ± 0.12
Ostase (*μ*g/L)	12.35 ± 3.63	13.82 ± 2.74	14.55 ± 4.63
PTH (ng/L)	40.71 ± 12.30	39.00 ± 10.77	33.94 ± 10.22
Postmenopausal ratio	10/10	16/16	33/36
Fasting blood glucose (mmol/L)	6.81 ± 1.13	8.37 ± 1.96*	8.06 ± 1.82*
Fasting insulin (*μ*IU/mL)	5.62 ± 2.99	5.52 ± 2.05	6.40 ± 3.46
HOMA-*β* (%)	36.15 ± 16.79	27.95 ± 20.06	35.26 ± 25.76
HOMA-IR	1.75 ± 1.23	2.04 ± 0.86	2.29 ± 1.42
tOC (ng/mL)	4.54 ± 0.75	4.20 ± 1.25	3.89 ± 0.81*
ucOC (pg/mL)	20.24 ± 11.70	20.05 ± 8.90	19.75 ± 9.77

The objects were divided into three groups, Group HbA1c-H (HbA1c ≥ 9%), Group HbA1c-M (7% ≤ HbA1c < 9%) and Group HbA1c-L (HbA1c < 7%).

*P < 0.05 when compared with Group HbA1c-L. ^#^ *P* < 0.05 when compared with Group HbA1c-M.

**Table 3:** Comparisons between Group tOC-H and Group tOC-L.

Parameters	All the objects		The male objects	
	tOC-H (*n* = 35)	tOC-L (*n* = 31)	tOC-H (*n* = 23)	tOC-L (*n* = 23)
Gender (female/male)	12/23	8/23	/	/
Age (year)	52.4 ± 11.5	51.2 ± 15.3	49.1 ± 11.9	50.2 ± 14.4
BMI (kg/m^2^)	24.57 ± 2.86	23.77 ± 3.31	24.58 ± 2.87	24.25 ± 3.06
Course of DM (month)	61.7 ± 51.2	72.9 ± 58.5	53.8 ± 47.4	59.6 ± 54.2
sCr (*μ*mol/L)	73.0 ± 11.5	72.3 ± 13.7	78.3 ± 9.8	76.4 ± 10.9
Ca (mmol/L)	2.40 ± 0.11	2.35 ± 0.14	2.40 ± 0.12	2.35 ± 0.15
Ostase (*μ*g/L)	15.59 ± 3.99	16.05 ± 2.67	14.50 ± 3.52	14.89 ± 2.44
PTH (ng/L)	36.99 ± 11.35	36.86 ± 11.03	36.87 ± 11.78	37.43 ± 11.61
Postmenopausal ratio	34/35	29/31	/	/
Fasting blood glucose (mmol/L)	7.95 ± 2.10	8.28 ± 1.80	7.61 ± 1.47	8.60 ± 1.73*
Fasting insulin (*μ*IU/mL)	6.34 ± 3.25	5.78 ± 2.75	6.21 ± 3.74	5.27 ± 2.50
HbA1c (%)	9.1 ± 2.3	10.0 ± 2.2	8.7 ± 2.2	10.0 ± 2.3
HOMA-*β* (%)	33.88 ± 20.44	30.30 ± 22.75	32.23 ± 16.81	23.83 ± 13.58
HOMA-IR	2.27 ± 1.40	2.08 ± 1.07	2.19 ± 1.63	2.01 ± 1.08

The objects were divided into two groups according to the average level of tOC, Group tOC-H and Group tOC-L.

*P < 0.05 when compared with Group tOC-H.

**Table 4:** Comparisons between Group ucOC-H and Group ucOC-L.

Parameters	All the objects		The male objects	
	ucOC-H (*n* = 24)	ucOC-L (*n* = 42)	ucOC-H (*n* = 16)	ucOC-L (*n* = 30)
Gender (female/male)	9/15	11/31	/	/
Age (year)	53.8 ± 12.8	50.8 ± 13.6	53.4 ± 12.1	47.8 ± 13.3
BMI (kg/m^2^)	23.55 ± 2.84	24.55 ± 3.17	22.93 ± 2.62	25.16 ± 2.84*
Course of DM (month)	78.6 ± 66.0	60.6 ± 47.1	64.7 ± 71.5	52.6 ± 36.4
sCr (*μ*mol/L)	70.6 ± 13.9	73.8 ± 11.6	78.9 ± 11.9	76.6 ± 9.4
Ca (mmol/L)	2.38 ± 0.14	2.38 ± 0.12	2.36 ± 0.15	2.38 ± 0.13
Ostase (*μ*g/L)	14.82 ± 3.82	13.50 ± 3.84	14.27 ± 3.28	13.00 ± 3.13
PTH (ng/L)	38.78 ± 10.89	39.15 ± 10.71	34.75 ± 13.06	38.34 ± 10.78
Postmenopausal ratio	22/24	41/42	/	/
Fasting blood glucose (mmol/L)	7.60 ± 2.27	8.37 ± 1.74*	7.38 ± 1.23	8.45 ± 1.74*
Fasting insulin (*μ*IU/mL)	5.49 ± 2.66	6.40 ± 3.19	4.67 ± 2.49	6.29 ± 3.40
HbA1c (%)	9.9 ± 2.5	9.3 ± 2.2	9.7 ± 2.8	9.2 ± 2.1
HOMA-*β* (%)	35.37 ± 27.83	30.55 ± 17.23	25.65 ± 13.29	29.36 ± 16.89
HOMA-IR	1.85 ± 1.04	2.36 ± 1.33	1.59 ± 1.10	2.35 ± 1.44*

The objects were divided into two groups according to the average level of ucOC, Group ucOC-H and Group ucOC-L. *P < 0.05 when compared with Group ucOC-H.

**Table 5:** Stepwise multiple regression analysis showing variables independently associated with HbA1c.

	Independent variable	Regression coefficient	*P* value	95% C.I. of the regression coefficient
All the objects	Ostase	0.253	0.000**	0.121~0.386
	tOC	−1.054	0.000**	−1.624~−0.483
The male objects	Ostase	0.215	0.044*	0.007~0.424
	tOC	−1.108	0.005**	−1.853~−0.362

**P < 0.01, *P < 0.05. HbA1c was the dependent variable. C.I. represents confidence interval.
